# A Novel Evidence That Mannan Binding Lectin (MBL) Pathway of Complement Cascade Activation is Involved in Homing and Engraftment of Hematopoietic Stem Progenitor Cells (HSPCs)

**DOI:** 10.1007/s12015-020-09983-8

**Published:** 2020-05-14

**Authors:** Mateusz Adamiak, Monika Cymer, Krzysztof Anusz, Michał Tracz, Mariusz Z. Ratajczak

**Affiliations:** 1 Center for Preclinical Studies and Technology, Department of Regenerative Medicine, Medical University of Warsaw, Warsaw, Poland; 2 Institute of Veterinary Medicine, Department of Food Hygiene and Public Health Protection, Warsaw University of Life Sciences (WULS-SGGW), Warsaw, Poland; 3 Stem Cell Institute at James Graham Brown Cancer Center, University of Louisville, 500 S. Floyd Street, Rm. 107, KY 40202 Louisville, USA

## Abstract

Delayed homing and engraftment of hematopoietic stem progenitor cells (HSPCs) or even failure to engraft at all is significant clinical problem after hematopoietic transplant. Therefore, in order to develop more efficient homing and engraftment facilitating strategies it is important to learn more about this process. Our team has postulated that myeloablative conditioning for transplantation induces in bone marrow (BM) microenvironment a state of sterile inflammation in which elements of innate immunity activated by radio- or chemotherapy conditioning for transplant play an important role. In frame with this claim we reported that a significant role in this process plays activation of complement cascade (ComC). Accordingly, mice that that lack a fifth component (C5) of ComC turned out to engraft poorly with normal syngeneic BM cells as compared to normal control animals. In extension of our previous studies we provide for first time evidence that mannan binding lectin (MBL) pathway is involved in activation of ComC in myeloablated transplant recipient BM and thus plays an important role in homing and engraftment of HSPCs. To support this MBL-KO mice show significant defect in hematopoietic reconstitution after hematopoietic transplantation. This correlates with a decrease in expression of stromal derived factor-1 (SDF-1) and impaired activation of Nlrp3 inflammasome in irradiated BM of these mice.

## Introduction

Hematopoietic transplants since more than 50 years are established the most successful therapeutic application of stem cells. Hematopoietic stem/progenitor cells (HSPCs) present in harvested from a donor bone marrow (BM), mobilized peripheral blood (mPB) or umbilical cord blood (UCB) unit are infused intravenously into myeloablated patient to home and subsequently engraft and expand in recipient BM microenvironment, with aim to establish long term normal hematopoiesis [1–4].

A first step in this process is homing of infused intravenously HSPCs to BM followed by their lodging into hematopoietic niches and engraftment. The homing process is facilitated by chemoattractants secreted from BM in response to myeloablative conditioning [4–6]. A crucial role in this step plays an α-chemokine stromal derived factor-1 (SDF-1) secreted by BM cells that survived myeloablative treatment [5, 7, 8]. Since HSPCs express on surface a functional receptor for SDF-1, seven transmembrane receptor G_αi_-protein coupled CXCR4 receptor, SDF-1-CXCR4 axis directs their navigation to BM niches [7, 9]. A supportive role for SDF-1-CXCR4 axis in homing and engraftment play also bioactive phosphosphingolipids – spinhigosine-1 phosphate (S1P) and ceramide-1 phosphate (C1P) as well extracellular nucleotide adenosine triphosphate (eATP) [5, 10]. All these homing factors are upregulated in parallel with SDF-1 in myeloablated by radio/chemotherapy BM [11].

Mounting evidence shows that myeloablative treatment of hematopoietic transplant donor induces state of “sterile inflammation” in BM microenvironment [12, 13]. This process is triggered by activation of radio- chemotherapy resistant macrophages, bone marrow stroma cells and complement cascade (ComC) [11, 14]. To support this our previous work demonstrated that mice deficient in the fifth element of ComC (C5-deficient mice) engraft worse with syngeneic BM cells as compared to control animals [11]. The ComC is activated by three crucial pathways known as (i) classical-, (ii) mannan binding lectin (MBL) - and (iii) alternative pathway [15, 16]. In particular MBL pathway of ComC activation is triggered by several danger associated molecular pattern molecules (DAMPs) or alarmines that are released from BM residing cells in response to sterile inflammation [17–22].

In our previous work we reported that MBL pathway of ComC activation plays an important role in pharmacological mobilization of HSPCs [23]. Herein, we asked if it may also play a role in their homing and engraftment. To address this question we employed MBL-deficient mice as a model to study its role in ComC activation after myeloablative conditioning for transplant and to assess a role of MBL in homing and engraftment of transplanted BM cells.

Our data demonstrates for a first time that MBL-deficient mice engraft worse with normal HSPCs as compared to control animals and this defect correlates with decreased induction of sterile inflammation in BM tissue as evidenced by decreased expression of SDF-1, activation of Nlrp3 inflammasome, and release of several DAMPs including extracellular adenosine triphosphate (eATP), high mobility group box 1 protein (HMGB-1) and S100 calcium-binding protein A8 and A9 (S100A8/9), that activate ComC. Therefore, we provide further evidence on a role of MBL-ComC axis as mediator of BM sterile inflammation in response to myeloablative conditioning and its role in homing and engraftment of HSPCs.

## Materials and Methods

### Animals

Pathogen-free, 6-8-week-old C57BL/6J wild-type (WT) and B6.129S4-Mbl1^tm1Kata^ Mbl2^tm1Kata^/J (Mbl-KO) female mice were purchased from the Jackson Laboratory (Bar Harbor, ME; USA) at least 2 weeks before experiments. Animal studies were approved by the Animal Care and Use Committee of the Warsaw Medical University (Warsaw, Poland) and University of Louisville (Louisville, KY, USA).

### Transwell Migration Assay

650 µl RPMI-1640 medium with 0.5% BSA alone or containing SDF-1 (5 ng/ml), or conditioned medium harvested from BMMNCs of irradiated (1000 cGy) WT or Mbl-KO mice was added to the lower chambers of a Costar Transwell 24-well plate (Corning Costar). Aliquots of cell suspension (1 × 10^6^ cells per 100 µl) were loaded onto the upper chambers with 5-µm pore filters and then incubated for 3 h (37 °C, 5% CO_2_). An aliquot of cells from the lower chambers was harvested and divided into two groups. I group was counted by FACS analysis. The cells were gated according to their forward scatter (FSC) and side scatter (SSC) parameters and counted during a 30-s acquisition at a high flow rate. The II group were resuspended in human methylcellulose base medium (R&D Systems), supplemented with murine GM-CSF (25 ng/ml) and IL-3 (10 ng/ml), for determining the number of CFU-GM colonies. Cultures were incubated for 7 days (37 °C, 95% humidity, and 5% CO_2_), at which time the colonies were counted under an inverted microscope [24–26].

### Short-term Homing Experiments

WT and Mbl-KO mice were irradiated with a lethal dose of γ-irradiation (10 Gy). Twenty-four hours after irradiation, the animals were transplanted (by tail vein injection) with 5 × 10^6^ BM cells from WT mice labeled with PKH67 Green Fluorescent Cell Linker (Sigma-Aldrich, St Louis, MO, USA) according to the manufacturer’s protocol. At 24 h after transplantation, BM cells from the femurs were isolated via Ficoll-Paque and divided. A part of the cells were analyzed on a flow cytometer. The rest of the cells were plated in serum-free methylcellulose cultures and stimulated to grow CFU-GM colonies with granulocyte-macrophage colony-stimulating factor (GM-CSF, 25 ng/ml) and interleukin 3 (IL-3, 10 ng/ml). After 7 days of incubation (37 °C, 95% humidity, and 5% CO_2_) the number of colonies was scored under an inverted microscope [25, 27].

### Evaluation of Engraftment

For engraftment experiments, WT and Mbl-KO mice were irradiated with a lethal dose of γ-irradiation (10 Gy). Twenty-four hours after irradiation, mice were transplanted with 1.5 × 10^5^ BM cells from WT mice by tail vein injection. 12 days after transplantation, femora of transplanted mice were flushed with phosphate-buffered saline (PBS). BM cells purified via Ficoll-Paque were plated in serum-free methylcellulose cultures and stimulated to grow CFU-GM colonies with G-CSF (25 ng/ml) and IL-3 (10 ng/ml). After 7 days of incubation (37 °C, 95% humidity, and 5% CO_2_) the number of colonies was scored under an inverted microscope. Spleens were also removed, fixed in Telesyniczky’s solution for CFU-S assays, and colonies were counted on the surface of the spleen [25, 27, 28].

### Recovery of Leukocytes and Platelets

For transplantation experiments, mice were irradiated with a lethal dose of γ-irradiation (10 Gy). 24 h later, mice were transplanted by tail-vein injection with 7.5 × 10^5^ BM cells. Transplanted mice were bled at various intervals from the retro-orbital plexus to obtain samples for white blood cell (WBC) and platelet (PLT) counts as described [25, 27, 29]. Briefly, 50 µl of PB were taken into EDTA-coated Microvette tubes (Sarstedt Inc., Newton, NC, USA) and run within 2 h of collection on a HemaVet 950FS hematology analyzer (Drew Scientific Inc., Oxford, CT, USA).

### Real-time Quantitative Reverse-transcription PCR

WT and Mbl-KO mice were irradiated with a lethal dose of γ-irradiation (10 Gy). Twenty-four hours after irradiation total RNA of murine BM-MNCs was isolated with the RNeasy Kit (Qiagen, Valencia, ca., USA). The RNA was reverse-transcribed with MultiScribe reverse transcriptase and oligo-dT primers (Applied Biosystems, Foster City, ca., USA). Quantitative assessment of mRNA levels was done by real-time RT-PCR using an ABI 7500 instrument with Power SYBR Green PCR Master Mix reagent. PCR conditions were as follows: 95 °C (15 s), 40 cycles at 95 °C (15 s), and 60 °C (1 min). According to melting point analysis, only one PCR product was amplified under these conditions. The relative quantity of a target, normalized to the endogenous β2 microglobulin gene as control and relative to a calibrator, is expressed as 2^− DDCt^ (fold difference), where Ct is the threshold cycle, DCt = (Ct of target genes) − (Ct of the endogenous control gene, β-microglobulin), and DDCt = (DCt of samples for the target gene) − (DCt of the calibrator for the target gene). The following primer pairs were used for analysis:

- β2m
- 5′-ATGCTATCCAGAAAACCCCTCAAAT-3′ (forward)
- 5′-AACTGTGTTACGTAGCAGTTCAGTA-3′ (reverse)
- Nlrp3
- 5′- ACCAGCCAGAGTGGAATGAC − 3′ (forward)
- 5′- ATGGAGATGCGGGAGAGATA − 3′ (reverse)
- Asc
- 5′- GCCAGAACAGGACACTTTGTG − 3′ (forward)
- 5′- AGTCAGCACACTGCCATGC − 3′ (reverse)
- Casp1
- 5′- GCTTTCTGCTCTTCAACACC − 3′ (forward)
- 5′- AAAATGTCCTCCAAGTCACAAG − 3′ (reverse)
- SDF-1
- 5′-CGT GAG GCC AGG GAA GAG T-3′ (forward)
- 5′-TGA TGA GCA TGG TGG GTT GA-3′ (reverse)
- IL-1β
- 5′- AGTTGACGGACCCCAAAAG − 3′ (forward)
- 5′- CTTCTCCACAGCCACAATGA − 3′ (reverse)
- IL-18
- 5′- ACAACTTTGGCCGACTTCAC − 3′ (forward)
- 5′- GTCTGGTCTGGGGTTCACTG − 3′ (reverse)
- NRLP1a
- 5′- AGGTGGAGCTAATGAAGCACA − 3′ (forward)
- 5′- CCATGTTAGAAGAGGGTAAAGAGC − 3′ (reverse)
- NRLP1b
- 5′- AGAGGTGGAGCTGATGAAGC − 3′ (forward)
- 5′- ACCATGTGGGGTCCAGAGT − 3′ (reverse)

### Enzyme-Linked Immunosorbent Assay and Measuring of ATP Levels

WT and Mbl-KO mice were irradiated with a lethal dose of γ-irradiation (10 Gy). Twenty-four hours after irradiation murine blood was obtained from the vena cava (1-ml syringe containing 100 µl of 0.5M EDTA). Plasma samples were prepared by taking the top fraction after centrifugation at 600 × g for 10 min at 4 °C and immediately freezing at − 80 °C. The residual C5a, IL-1β, IL-18 and HMGB1 level were measured by enzyme-linked immunosorbent assay (ELISA) according to the manufacturer’s protocols (Cloud-Clone). The ATP levels were measured using the ATP Colorimetric/Fluorometric Assay kit and the Deproteinizing Sample Preparation kit (BioVision, Milpitas, ca., USA), according to the manufacturer’s protocol. Fluorescence analysis was performed with Ex/Em set at 535/585 nm. Results are presented as % of control [15, 30].

### Statistical Analysis

All results are presented as mean ± SD. Statistical analysis of the data was done using Student’s t-test for unpaired samples, with p ≤ 0.05 considered significant.

## Results

### Decrease in Chemotactic Activity of Conditioned Media Isolated From BM From Irradiated MBL-KO Mice

**Fig. 1:**
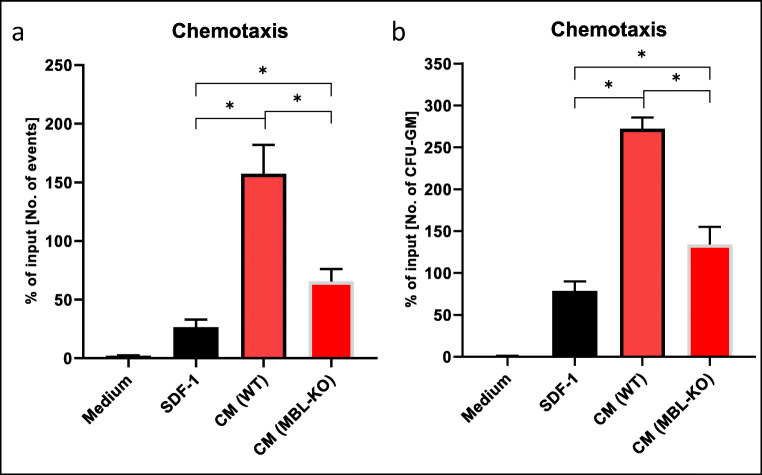
Impact of MBL deficiency on chemotactic activity of murine BMMNCs. The chemotactic responsiveness of BMMNCs to medium alone or supplemented with SDF-1 or conditioned medium harvested from BMMNCs of irradiated (10 Gy) WT or Mbl-KO mice evaluated by FACS to enumerate cell number in lower Transwell chambers (**a**) and by clonogenic in vitro assays with CFU-GM progenitors recovered from lower Transwell chambers (**b**). Results are combined from two independent experiments performed in triplicate and shown as a percent of input (5% of insert). *p < 0.05

It has been reported that myeloablative conditioning for hematopoietic transplantation upregulates several chemoattractants in BM microenvironment including stromal derived factor-1 (SDF-1), sphingosine-1 phosphate (S1P), and extracellular ATP (eATP) [7, 8, 11]. Based on this data we have collected, conditioned medium from cells isolated from BM of irradiated MBL-KO mice and normal animals and performed Transwell chemotactic assays with normal wild type BM cells. Figure 1 shows that CM medium from MBL-deficient mice has reduced by more than 50% chemotactic activity as compared to CM harvested from BM of irradiated normal control mice. Both the number of BMMNC (Fig. 1a) and CFU-GM clonogeneic progenitors (Fig. 1b) that responded to CM was reduced when MBL-KO mice BM CM was placed in lower chambers of Tranwells. This data indicates that CM from irradiated MBL-KO mice has lower chemotactic potential for HSPCs.

### MBL-deficient Mice Display Decreased Homing and Engraftment of Infused Normal Mice BM cells

**Fig. 2:**
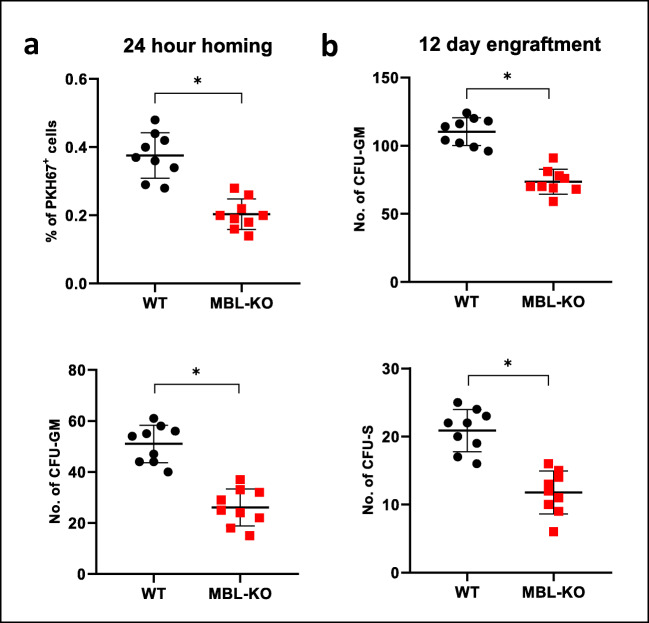
Defect in homing and short-term engraftment of WT HSPCs in Mbl-KO mice.** a **24 h homing. Lethally irradiated mice (9 per group) were transplanted with bone marrow mononuclear cells (BMMNCs) from WT mice, labeled with a PKH67 cell linker. Twenty-four hours after transplantation, femoral BMMNCs were harvested, the number of PKH67 cells was evaluated by FACS (upper part of panel A), and the clonogenic CFU-GM progenitors were enumerated in an *in vitro* colony assay (lower part of panel A). **b **12 days engraftment. Lethally irradiated mice (9 per group) were transplanted with bone marrow mononuclear cells (BMMNCs) from WT mice, and 12 days after transplantation femoral BMMNCs were harvested and plated to enumerate the number of growing CFU-GM colonies (upper part of panel B) and spleens were removed for counting the number of CFU-S colonies (lower part of panel B). No colonies were formed in lethally irradiated or nontransplanted mice (irradiation control). *p < 0.05

Next we performed transplantation studies with normal syngeneic BM cells into lethally irradiated MBL-deficient and normal control mice. Homing was evaluated by number of fluorochrome PKH67 labeled cells detected in BM of transplanted mice and number of CFU-GM clonogeneic progenitors derived from donor BM cells. As it is demonstrated in Fig. 2a, the MBL-KO mice show a 50% reduction of BM homing of transplanted HSPCs. Similarly, we observed defective early 12 days engraftment of HSPCs in BM of MBL-deficient mice as assayed by evaluating presence of clonogeneic CFU-GM in BM of transplanted mice after 12 days as well as the number of 12 days spleen colonies (ang. Colony forming units in spleen; CFU-S) after injection of syngeneic BM cells **(**Fig. 2b**)**.

**Fig. 3:**
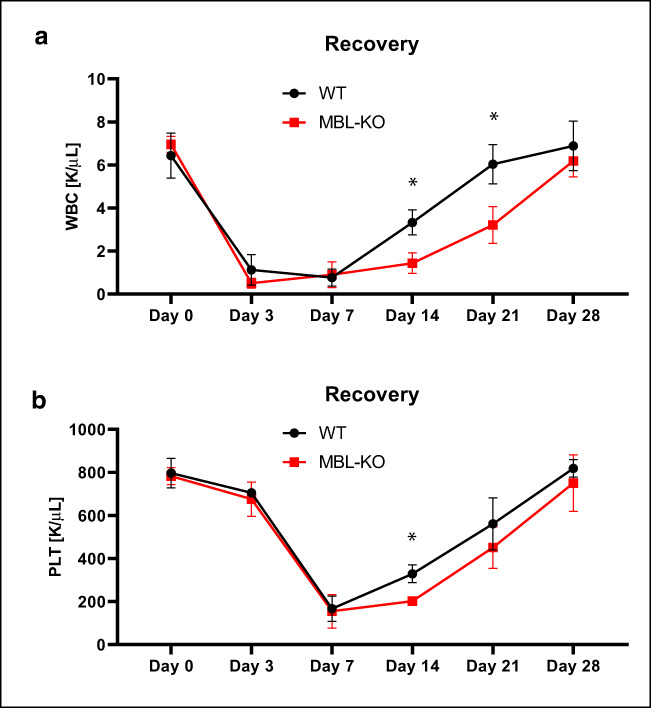
A defect in long-term engraftment of WT HSPCs in Mbl-KO mice. Lethally irradiated mice (9 per group) were transplanted with bone marrow mononuclear cells (BMMNCs) from WT mice. White blood cells (**a**) and platelets (**b**) were counted at intervals (at 0, 3, 7, 14, 21, and 28 days after transplantation). *p < 0.05

To focus better on this phenomenon we transplanted lethally irradiated MBL-deficient and control mice with syngeneic BM cells and followed recovery of peripheral blood counts. Figure 3a and b shows that MBL-deficient mice have delayed by 4–7 days recovery of peripheral blood leucocytes and platelets counts.

Based on this data MBL-KO mice display statistically significant defect in both homing (Fig. 2A) and engraftment of transplanted HSPCs (Figs. 2b and 3).

### Decreased Expression of SDF-1, Nlrp3 Inflammasome Components and Decrease in ComC Activation in MBL-KO Mice

**Fig. 4:**
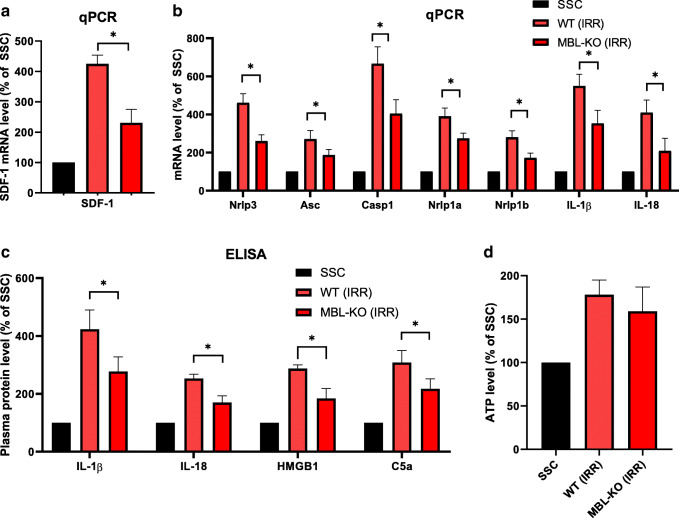
Impact of Mbl deficiency on sterile inflammation markers.** a** SDF-1 mRNA expression was evaluated at the mRNA level by real-time PCR. **b** Sterile inflammation genes expression was evaluated at the mRNA level by real-time PCR. **c** The level of IL-1β, IL-18, Hmgb-1 and C5a proteins in mouse plasma after irradiation measured by ELISA. **d** The ATP level was evaluated in plasma of irradiated WT and MBL-KO mice by employing ELISA. Results are shown as a percentage of control mice in steady state condition. Results from three independent experiments are pooled together. *p ≤ 0.05

To explain this data at molecular level we analyzed in conditioned BM the expression of selected factors playing an important role in homing and engraftment of HSPCs. Figure 4a shows difference in expression of mRNA for SDF-1 in BM extracts from irradiated by 1000 cGy MBL-KO and control mice as compared to non-irradiated control mice. As it is shown, mRNA for SDF-1 was decreased by 50% in BM from conditioned by irradiation MBL-KO animals as compared to control irradiated animals. We also observed decrease in expression of selected DAMPs such as HMGB1 and S100A8/9.

Next, since Nlrp3 inflammasome as we have demonstrated plays an important role in homing and engraftment of HSPCs, we evaluated expression of mRNA for Nlrp3 inflammasome components. Figure 4b shows that expression of mRNA for Nlrp3, ASC, caspase-1, Nrlp1a, Nrlp1b as well as IL-1β and IL-18 was expressed at much lower level in BM cells from irradiated MBL-KO mice as compared to WT animals. This indicates that activated ComC and its cleavage fragments including C3a, C5a and C5b-C9 (membrane attack complex, MAC) play an important role in maintaining and amplifying activation of Nlrp3 inflammasome in innate immunity cells [31, 32]. Interestingly, we also noticed a decrease in expression of mRNA for Nlrp1 and Aim2 inflammsomes.

We also performed analysis at protein level by employing sensitive ELISA assays (Fig. 4c). We confirmed higher activation of Nlrp3 inflammasome in plasma of irradiated WT mice by detecting higher level of IL-1β and IL-18 as compared to MBL-KO mice. We also detected higher expression of HMGB1 protein in plasma of irradiated WT mice and higher activation of ComC as measured by elevated level of C5a in WT mice plasma. At the same time we did not detect significant difference in level of extracellular ATP between plasmas from irradiated WT and MBL-KO mice **(**Fig. 4d**)**.

## Discussion

The mechanisms that regulate homing and engraftment of transplanted HSPCs are still not very well understood. It has been proposed that there is a tug of war over the chemotactic stromal derived factor-1 (SDF-1) gradient between irradiated BM and PB for HSPCs to home to the BM microenvironment [26, 33, 34]. SDF-1, which binds to the G_αi_ protein-coupled, seven-transmembrane-spanning CXCR4 receptor highly expressed on HSPCs, plays an unquestioned role in developmental migration of HSPCs during embryogenesis, their retention in the postnatal BM microenvironment, and homing after hematopoietic transplantation [7, 9].

Herein, we postulate that sterile inflammation after myeloablative conditioning for transplantation orchestrates homing and engraftment of transplanted HSPCs. Our recent research indicates that an important role in its induction in irradiated BM plays the activation of Nlrp3 inflammasome in cells relatively more resistant to myeloablative conditioning including (i) irradiation resistant macrophages, (ii) BM stroma cells, and (iii) endothelial cells. In fact, we noticed that mRNA for Nlrp3 inflammasome components were upregulated at higher level in BM cells isolated from control normal mice as compared to MBL-KO animals. One of the markers of activation of Nlrp3 inflammasome is increase in plasma level of IL-1β and IL-18 that are secreted from the cells in Nlrp3 inflammasome activation-dependent manner [35, 36]. In agreement with mRNA data, we also detected higher expression of both cytokines at protein level by sensitive ELISA in plasma of control mice as compared to plasma from MBL-KO animals. Moreover, we noticed decrease in expression of mRNA for important DAMPs or alarmines in BM cells including HGMB1 and S100A8/9 in MBL-KO mice BM cells. This decrease in expression of Nlrp3 components as well as DAMPs could be explained by decrease in activation of ComC and release of C3a, C5a and C5b-C9 (MAC) complement cleavage products in MBL-KO mice, that are needed to maintain and amplify activation of Nlrp3 inflammasome in BM cells [23, 37, 38]. In fact, as expected, activation of ComC as represented by plasma level of C5a anaphylatoxin was reduced in MBL-KO mice.

In our previous work we have demonstrated that activation of ComC in BM microenvironment after conditioning for hematopoietic transplant plays an important role in homing and engraftment of HSPCs [11]. Accordingly, mice deficient in fifth component of ComC – C5a-deficient displayed after transplantation with normal BM cells impaired homing and engraftment of HSPCs and delayed recovery of peripheral blood cell counts and hematopoietic reconstitution [11]. Herein, we became interested at molecular level how ComC is activated.

As mentioned, ComC may become activated by three pathways including classical-, alternative- and mannan binding lectin (MBL) pathway [15, 16]. It is known that the classical complement pathway typically requires for activation antigen-antibody immune complexes, the alternative pathway can be activated by C3 hydrolysis, foreign material, pathogens, or damaged cells, and MBL pathway recognizes carbohydrate patterns, found on the surface of bacteria, viruses, protozoa and fungi. In addition, all these pathways recognize own apoptotic cells as well as some DAMPs or alarmines released from damaged cells.

Conditioning for transplantation activates ComC as we have shown in the past [11] and confirm here again shown by increase in expression of C5 cleavage fragment C5a plasma level in control mice, and its lower level in MBL-KO mice. Therefore, we focused on a potential role of MBL pathway in ComC activation in myeloablated BM and our data indicate that this pathway plays an important and underappreciated role in sterile inflammation mediated homing and engraftment of transplanted HSPCs. Our data indicate that MBL pathway of ComC activation plays an important role in priming BM microenvironment for hematopoietic transplant. Nevertheless, further studies are still needed to study if classical and alternative pathway are also involved in myeloablative conditioning of BM for hematopoietic transplant. This requires further investigation using appropriate KO mice models.

What is important we noticed that conditioned medium from BM cells isolated from MBL-KO has decreased chemotactic activity against normal bone marrow mononuclear cells as well as against clonogeneic CFU-GM progenitors. This could be at least partially explained by decrease in expression of SDF-1 as well as other HSPCs chemoattractants in BM cells isolated from MBL-KO mice conditioned for transplant by irradiation. In fact, our data indicates a novel role of MBL pathway of ComC activation and induction of sterile inflammation that upregulates mRNA for SDF-1 in irradiated BM. We noticed that MBL-KO mice express mRNA for SDF-1 at much lower level than conditioned wild type animals. Nevertheless, we are aware that the homing process of HSPCs is supported also by gradient of S1P and eATP, and we have reported in the past that both S1P and eATP are upregulated in irradiated BM [11, 39]. In our current study, eATP level became upregulated in irradiated BM, but we did notice no significant difference between MBL-KO and control normal mice, which suggests that MBL pathway may differently regulate expression of SDF-1 and eATP. An open question remains if it is any difference in expression of S1P, and we are currently addressing this issue. Since MBL-KO mice were still be able to engraft with normal BMMNCs, this suggests involvement of other compensatory mechanisms that may somehow mitigate MBL deficiency.

In conclusion, we provide further evidence for an important role of ComC activation in BM after myeloablative conditioning for hematopoietic transplant, as orchestrator of HSPCs homing and engraftment. We also provide a novel evidence for a role of MBL-pathway of ComC activation in this process and postulate that modulation of innate immunity in BM microenvironment may open new opportunities to optimize transplant protocols.
